# Upsurge in echovirus 30 detections in five EU/EEA countries, April to September, 2018

**DOI:** 10.2807/1560-7917.ES.2018.23.44.1800537

**Published:** 2018-11-01

**Authors:** Eeva K Broberg, Benedetto Simone, Josep Jansa, the EU/EEA Member State contributors

**Affiliations:** 1 European Centre for Disease Prevention and Control, Stockholm, Sweden

**Keywords:** echovirus 30, enterovirus, epidemic, Europe

## Abstract

An upsurge in Echovirus 30 (E30) infections, associated with meningitis/meningoencephalitis, has been observed in Denmark, Germany, the Netherlands, Norway and Sweden in the period April to September 2018, compared with 2015–2017. In total, 658 E30 infections among 4,537 enterovirus infections were detected in 15 countries between January and September 2018 and affected mainly newborns and 26–45 year-olds. National public health institutes are reminded to remain vigilant and inform clinicians of the ongoing epidemic.

## Event history and methods

The Norwegian and Dutch national public health institutes observed an upsurge in the number of enterovirus positive detections, especially Echovirus 30 (E30) cases in July 2018 [1,2]. Pursuant to these findings, the European Centre for Disease Prevention and Control (ECDC) launched a European Union (EU)-wide call to report E30 cases through the Epidemic Intelligence Information System–Vaccine Preventable Diseases (EPIS-VPD) as there is no routine data collection on non-polio enteroviruses at European level. However, national-level laboratory-based non-polio enterovirus surveillance with typing exists in 26 countries in the EU and European Economic Area (EEA) [3]. The first data call was sent out on 20 July, the second on 3 August, and updates for August and September data were received by 22 October 2018. 

The aim of the study was to better understand the extent, severity and trend of the E30 epidemic in the EU/EEA in comparison with data from 2015 to 2017. Aggregate data were collected on the number of enterovirus and E30 detections per month, demographic information by age group, sex and clinical presentation. The call also asked for details on surveillance and typing methods and whether the data covered the whole country and was representative of the entire population. The EU/EEA country-specific E30 detections for the period from 2015 to 2017 were extracted from an unpublished ECDC survey (data not shown).

## Surveillance and typing methods in responding countries

Of 31 EU/EEA countries, 15 (Austria, Belgium, Croatia, Czech Republic, Denmark, Finland, Germany, Iceland, Latvia, the Netherlands, Norway, Slovenia, Spain, Sweden and the United Kingdom (UK) (England, Scotland and Wales)) responded to the data call, reporting 4,537 enterovirus-positive patients (Table 1). Eleven countries collected data from the whole country and 10 reported the data to be representative of the population (Table 1). The surveillance systems for E30 detections varied across the countries and were passive in 13 countries [4]. Twelve countries used viral protein (VP) 1 for genotyping, with the majority (n = 9) using the typing assay recommended by the World Health Organization (WHO) [5]. One country used antibody binding, one a neutralisation assay and one sent the positive specimens for genotyping to a reference laboratory (Table 1). 

**Table 1:** Type of data and number of enterovirus and echovirus 30 infections reported by country in the EU/EEA, January–September, 2018 (n = 4,537)

Country	Number of EV-positive patients	Number of E30 patients	Age reported for E30 patients	Sex reported for E30 patients^a^	Symptoms reported for E30 patients (CNS symptoms or detailed symptoms or no data)	Are the data from the whole country?	Are the data representative for the whole population?	Type of surveillance system: active or passive^b^	Type of reporting: voluntary reporting or notifiable disease	Typing protocol used	Comments on data collection or surveillance system
Austria	32 (missing data for July–September)	8 (missing data for August–September)	Yes	Yes	Detailed symptoms	No	Unknown	Passive	Voluntary	VP1 genotyping [5]	Performance of AFP and EV surveillance in Austria: All laboratories performing EV diagnostics recorded. These laboratories are required to submit their data quarterly or at the end of the year to the Ministry and submit the EV PCR-positive samples to the NRC for Poliomyelitis for further investigations (typing). Available data are from Vienna, Upper Austria, Lower Austria, Carinthia, Burgenland and Styria from quarters 1 and 2 of 2018.
Belgium	57	13	Yes	Yes	Detailed symptoms	Yes	Unknown	Passive; active in case of outbreak	Voluntary	VP1 genotyping [28]	Data were reported by the NRC for EV. This NRC receives samples for viral sequencing from various laboratories on a voluntary basis. Results shown are those of EV-positive CSF samples. In addition to the NRC analyses and data collection, EV surveillance in Belgium is based on a network of sentinel laboratories reporting EV-positive CSF. However, EV typing is not reported by this network as sequencing is not systematically performed.
Croatia	18	3	Yes	Yes	Detailed symptoms	Yes	Yes	Passive	Voluntary	Typing by direct antibody (echovirus antibody test, Light Diagnostics, EMD Millipore Corp., United States) and neutralisation as confirmatory assay	Positive, untypable specimens are sent to the reference laboratory Helsinki, Finland (see entry for Finland below) for sequencing.
Czech Republic	147	0	NA	NA	NA	Yes	Yes	Passive; active following detection of a case	Notifiable	VP1 genotyping [5]	The Czech Republic has a mandatory/notifiable reporting system of infectious diseases (ISIN) including EV with a passive reporting system for most of them.
Denmark	402	95	Yes	Yes	CNS symptoms	Yes	Yes	Passive	Voluntary	VP1 [5] and VP4/VP2 [29] genotyping	Some genotyping results are still pending for August and September 2018. Clinical data are not available for the majority of cases at this date. CNS symptoms are assumed when CSF is the sample material.
Finland	232	1 (missing data for August–September)	Yes	Yes	CNS symptoms	Yes	Yes	Passive	Notifiable	VP1 genotyping [5]	EV laboratory findings are notifiable, the disease not. The data are collected by the National Institute for Health and Welfare. Typing is done mostly in the University Hospitals in Helsinki and Turku. Most EVs typed from January to July, typing results are pending for August and September 2018.
Germany	320	80	Yes	Yes	CNS symptoms	Yes	No	Passive	Voluntary	VP1 genotyping [12,30]	Some typing results are still pending for August and September 2018. Samples from hospitalised patients with suspected aseptic meningitis/encephalitis or AFP are sent for EV testing to the Laboratory Network for EV Diagnostics.
Iceland	28	5	Yes	Yes	CNS symptoms	Yes	Yes	Passive	Voluntary	Typing performed in reference laboratory in Helsinki, Finland (see above)	All virological specimens are received in one laboratory in the country.
Latvia	157	8	Yes	Yes	Detailed symptoms	Yes	Yes	Passive	Voluntary	Typing by neutralisation assay	Detailed symptoms are reported but the symptoms of the reported cases were unknown. Surveillance system is based mainly on hospital samples.
The Netherlands	Not available (488 EV typed)	152	Yes	Yes	Detailed symptoms	No	Yes	Passive	Voluntary	VP1 genotyping [5]	The VIRO-TypeNed working group only collect data on EV types. Denominators such as number of tested and number of EV-positives are collected once at the end of the year for the national certification committee/WHO.
Norway	735	68	Yes	Yes	Detailed symptoms	Yes	Yes	Active	Voluntary^c^	VP1 genotyping [5]	Only cases with CNS infections caused by EV are notified in the national surveillance system. Positive EV cases are reported from 14 of 19 laboratories throughout the country.
Slovenia	96	0	NA	NA	NA	Yes	No	Passive	Voluntary	VP1 genotyping [5]	The data are available from the Laboratory for Public Health Virology of the National Laboratory of Health, Environment and Food, which serves as WHO NRC for Polioviruses and as WHO National Influenza Centre for the country. Specimens from AFP surveillance, residual stool specimens of patients younger than 15 years from supplementary polio/EV surveillance and all nasal/throat swabs from ILI/ARI surveillance are tested for presence of EV (molecular detection in all cases). A proportion of positive specimens are typed. A limited number of clinical laboratories in the country also perform EV molecular detection in suspected cases; typing is not performed routinely for these cases; these data are not available at the moment.
Spain	414	38	Yes	Yes	Detailed symptoms	No	Yes	Passive	Voluntary	VP1 genotyping [31]	Data were reported by the Spanish EV Reference Laboratory. The laboratory receives EV-positive samples for poliovirus exclusion and genotyping from many hospital laboratories throughout the country on a voluntary basis.
Sweden	Not available (247 EV typed)	75	Yes	Yes	No	Yes	Yes	Passive	Voluntary; EV meningitis is notifiable	VP1 genotyping [5]	Typing data are available from the Public Health Agency of Sweden (PHAS) which is the Swedish WHO NRL for poliovirus. PHAS has been conducting supplementary EV surveillance for many years, which is based on isolation and typing of EV in (mainly) stool samples from patients with verified EV-associated meningitis.
United Kingdom^d^	1,164 (England, Scotland and Wales; August–September counts only for typed viruses in England)	112	Yes (no data for Wales)	Yes	Detailed symptoms (no data for Wales)	No (England, Scotland and Wales)	Unknown	Active	Voluntary	VP1 genotyping [5]	England: EV detections by NHS laboratories are notified electronically through Public Health England Second Generation Laboratory Surveillance System. As part of the enhanced poliovirus surveillance, microbiologists are reminded to send any untyped EV-positive samples from certain sample sites or symptoms for EV characterisation to the NRL. Characterisation is incomplete for August and September. Scotland: EV detections by NHS Laboratories are notified electronically to Health Protection Scotland through the Electronic Communication of Surveillance in Scotland.
**Total**	**4,537^e^**	**658**	**13 Yes**	**13 Yes**	**12 with CNS or detailed symptom data**	**11 Yes**	**10 Yes**	**13 passive**	**13 voluntary**	**12 VP1 genotyping**	

AFP: acute flaccid paralysis; ARI: acute respiratory infection; CNS: central nervous system; CSF: cerebrospinal fluid; EAA: European Economic Area; EU: European Union; E30: echovirus 30; EV: enterovirus; ILI: influenza-like illness; NA: not applicable; NHS: National Health Service; NRC: National Reference Center; NRL: National Reference Laboratory; PCR: polymerase chain reaction; WHO: World Health Organization.

^a^ Countries who report on the age, sex and detailed symptom data for enteroviruses, but did not have any E30 cases to report on, have been marked as NA.

^b^ An active surveillance system was defined as a system that is based on the public health officials' initiative to contact the physicians, laboratory or hospital staff or other relevant sources to report data. Passive surveillance was defined as a system relying on the physicians, laboratory or hospital staff or other relevant sources to take the initiative to report data to the health department [4].

^c^ Notifiable for CNS infections with enterovirus detection.

^d^ The data are from England, Scotland and Wales. Northern Ireland did not participate.

^e^ Total includes 735 typed enteroviruses from the Netherlands and Sweden.

## Reported cases in 2018

**Figure 1:**
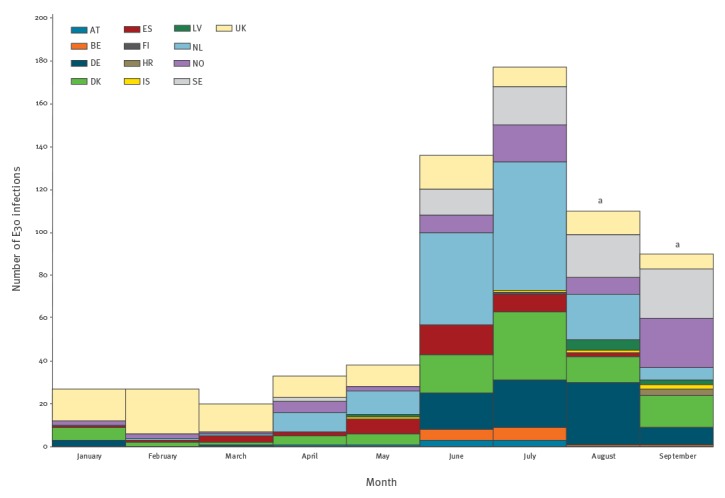
Number of echovirus 30-positive patients by month and countries reporting detections, 13 EU/EEA countries, January–September, 2018 (n = 658)

AT: Austria; BE: Belgium; DE: Germany; DK: Denmark; ES: Spain; EAA: European Economic Area; EU: European Union; E30: echovirus 30; FI: Finland; HR: Croatia; IS: Iceland; LV: Latvia; NL: the Netherlands; NO: Norway; SE: Sweden; UK: United Kingdom (England, Scotland and Wales).

^a^ Typing data were not complete for August and September at the time of publishing.

Thirteen countries (Austria, Belgium, Croatia, Denmark, Finland, Germany, Iceland, Latvia, the Netherlands, Norway, Spain, Sweden and the UK) reported 658 E30-positive patients to ECDC for the period from 1 January to 30 September 2018 (Table 1, Figure 1). The number of E30 detections started to increase after March, with 33 cases reported in April and 38 in May, and increased further to 136 in June and 177 in July (Figure 1), after which the numbers decreased. The Czech Republic and Slovenia had not detected any E30 infections in 2018 until the end of September.

See Table 1 for details on the countries. Austria reported data by quarter (n=3 in the period from April to June); for the purposes of this graph, we display them as 1 case per month in that quarter. Austria subsequently reported an additional two E30 infections for June, and therefore the total count for June is three. The Czech Republic and Slovenia did not detect any E30 between January and September 2018 and are therefore not included in the figure.

**Figure 2:**
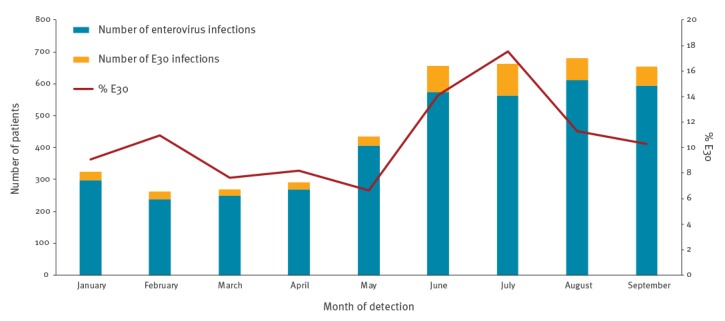
Number of echovirus 30 and other typed and untyped enterovirus infections and proportion of E30, by month of detection, 13 EU/EEA countries, January–September, 2018 (enterovirus infections: n = 3,802; E30 infections: n = 431)

EAA: European Economic Area; EU: European Union.

Twelve countries (Belgium, Croatia, Czech Republic, Denmark, Finland, Germany, Iceland, Latvia, Norway, Slovenia, Spain and the UK (England and Wales)) also submitted overall enterovirus detection data by month; Austria submitted enterovirus detections by quarter (Figure 2). The proportion of E30 detections among the enterovirus-positive samples was at its lowest in May (7%; 27/407 EVs), increased to 14% (81/575) in June and further to 18% (99/564) in July, and returned to 11% in August (69/611) (Figure 2). The proportion of E30 infections among typed enteroviruses in July was 37% for the Netherlands and 29% for Sweden. Those two countries submitted number of typed enteroviruses instead of total number of enterovirus-positive samples.

For the United Kingdom, the enterovirus infections were reported only by England and Wales. The Netherlands and Sweden did not provide any counts for enterovirus-positive detections but rather typing results and are therefore not included.

## Comparison of 2018 findings with data from 2015 to 2017

**Figure 3:**
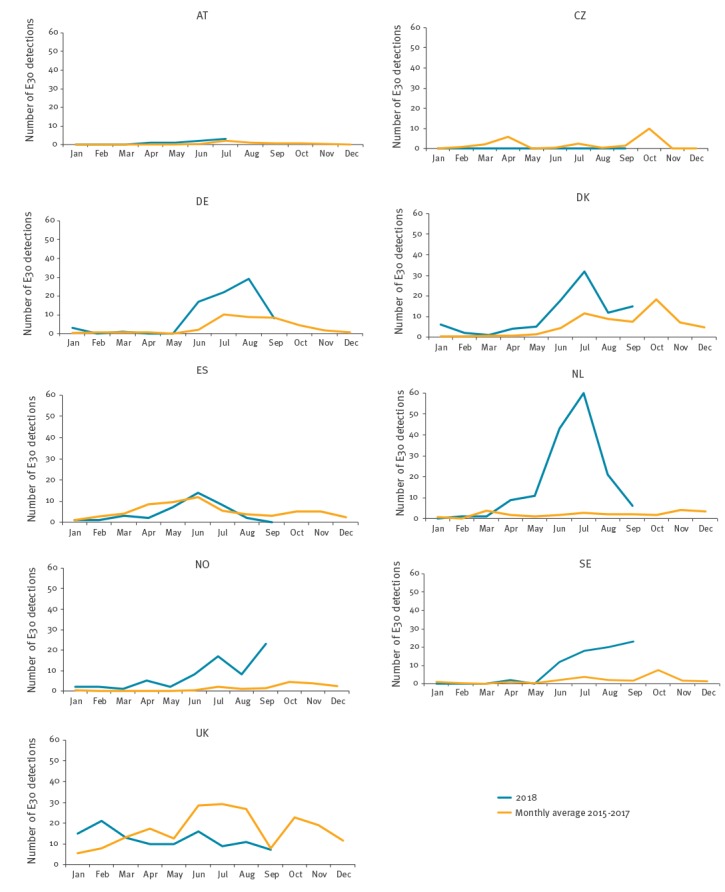
Number of detections of echovirus 30 in 2018 compared with mean number of detections per month in 2015–2017, by country, 9 EU/EEA countries, January–September, 2018 (n = 627)

AT: Austria; CZ: Czech Republic; DE: Germany; DK: Denmark; ES: Spain; EAA: European Economic Area; EU: European Union; NL: the Netherlands; NO: Norway; SE: Sweden; UK: United Kingdom (England, Scotland and Wales).

We compared the 2018 counts of E30 detections with data for the period 2015 to 2017, reported through an unpublished ECDC survey (data not shown). Data from a country were included if more than 10 E30 detections were reported for the period 2015 to 2017 and if the country responded to our data call. Nine of 15 participating countries were included in this analysis. Based on the available data, a more than twofold increase in detections was shown for Denmark from May to July and in September, for Germany from June to August, for the Netherlands from April to September, for Norway from July to September and for Sweden from June to September 2018, compared with the respective monthly average of E30 detections during 2015 to 2017 (Figure 3). Based on the data from 2015 to 2017, the UK experienced a marked increase of E30 detections in July and October 2017 that continued until February 2018, but has not observed an increase in summer 2018 (Figure 3). The Czech Republic experienced an increase in July 2016 and Denmark, the Netherlands, Norway and Sweden in October 2017.

Data were included if a country reported more than 10 E30 detections for 2015–2017.

The main age groups affected by the E30 infections in 2018 were newborns under the age of 3 months and adults aged 26–45 years (Table 2), although all age groups were affected. Of the 627 reported E30 cases with information on age group, 124 (20%) were newborns and 236 (38%) were adults aged 26–45 years. Ninety-two cases were aged 6–15 years, accounting for 15% of the cases. In the period from 2015 to 2017, E30 was also mostly observed in young adults (age group 26–45 years) and newborns up to 3 months of age; they represented, respectively, 35% and 19% of all E30 detected in the study period (data not shown).

**Table 2:** Echovirus 30 patients by age group, 14 EU/EEA countries^a^, January–September 2018 (n = 627)

Age group	Number of echovirus 30 patients	%
0–3 months	124	20
4–6 months	10	2
7–12 months	6	1
>1 year ≤5 years	53	8
6–15 years	92	15
16–25 years	79	13
26–45 years	236	38
46–65 years	20	3
>65 years	7	1
**Total**	**627**	**100**

EAA: European Economic Area; EU: European Union.

^a^ Reported by Austria, Belgium, Croatia, Czech Republic, Denmark, Finland, Germany, Iceland, Latvia, the Netherlands, Norway, Spain, Sweden and the United Kingdom (England and Scotland).

Sex was reported for 599 E30 patients and the male to female ratio was 1.4:1, with 353 cases reported as male.

## Central nervous system involvement

Cerebrospinal fluid (CSF) can be used as a proxy for central nervous system (CNS) symptoms as clinicians collect that specimen type mainly in the presence of neurological symptoms and on suspicion of infectious agent. Denmark reported that 53 (56%) of their 95 E30 detections were from CSF. Germany collects specimens from aseptic meningitis, encephalitis or acute flaccid paralysis cases only and therefore all 80 German E30 patients had symptoms compatible with CNS infection. Belgium also reported that all their 13 cases had meningitis symptoms. Information on clinical presentation was available from Austria, Belgium, Croatia, Finland, Iceland, Latvia, the Netherlands, Norway, Spain and the UK. Of the 277 E30 patients reported with clinical data, 208 (75%) had CNS symptoms. For 185 patients, further symptom categories were reported and those were classified as meningitis (n = 90; 49%), meningoencephalitis (n = 54; 29%), encephalitis (n = 1; <1%) or other CNS symptoms (n = 5; 3%). Thirty-five (19%) patients were recorded to have had other symptoms, mainly fever. No data were collected on recovery, mortality, nosocomial infections or co-detections.

## Discussion

From April to September 2018, an increase in E30 infections, associated with cases of meningitis and meningoencephalitis, has been detected in five countries in the EU/EEA, compared with previous years. 

E30 is an enterovirus of the B species that causes aseptic meningitis, often associated with outbreaks, some of which have been large [6-10]. Aseptic meningitis is the most commonly reported syndrome associated with E30 infections [8-11]. Although non-polio enterovirus infections are usually benign and self-limiting, they can also cause more severe, life-threatening diseases (e.g. encephalitis, paralysis, myopericarditis and neonatal enteroviral sepsis) [7]. The majority of the patients recover with symptomatic treatment within one week.

E30 epidemics occur usually as repeated cycles of emerging and dominating virus lineages that cause outbreaks every 3–5 years, often covering large geographical areas, and disappear thereafter to re-emerge later [12]. In the United States, E30 has caused outbreaks at irregular intervals but with a duration of several years [13]. In Europe, several countries have in recent years reported increased incidence of E30: Austria in 2000 [14], Spain from 2000 to 2002 [15], France in 2005 [16], Germany in 2008 [17], Finland from 2009 to 2010 [18,19], Latvia and Serbia in 2010 [20,21], Greece in 2012 [22] and France and Germany in 2013 [17,23]. In these outbreaks, E30 was detected mainly in children younger than 15 years [9,21-23] but also in adults [8,13]. Enteroviruses usually spread through person-to-person transmission via the faecal-oral or oral-oral route. However, echovirus outbreaks associated with swimming pools [24,25] as well as nature-like ponds [11] have been reported.

Based on the available data, five EU/EEA countries (Denmark, Germany, the Netherlands, Norway and Sweden) experienced or are still experiencing an E30 upsurge during summer and autumn 2018 compared with the same months of previous years. The current upsurges affected in particular newborns and adults aged 26–45 years of both sexes but with a predominance of males. The majority (75%) of reported patients experienced CNS symptoms, mainly meningitis or meningoencephalitis. In at least two of these countries, detection of upsurges and reporting of cases was particularly focused on patients with CNS symptoms and such cases are therefore likely to be over-represented. 

When interpreting the data, the following limitations have to be considered: firstly, enterovirus surveillance is mainly passive and therefore captures primarily viruses isolated from severe cases rather than from patients with mild diseases. This may skew the results towards severe outcomes even in the countries that have access to clinical information. Secondly, only 15 EU/EEA countries took part in the study and the data are therefore not representative for the whole EU/EEA region. Four countries also indicated that their data were not from all parts of the country and five that the data were not representative for the entire population or that representativeness was unknown. We also acknowledge that the level of testing and methods used for diagnostics and typing vary between the countries and may affect the sensitivity of detection and typing. Thirdly, some E30 results may have been missed with the regularly used VP1 sequence typing protocol [5] because of low viral load in the initial specimen, especially if CSF or serum was sent to the laboratory without an additional stool specimen, which is preferred. It also needs to be considered that some typing data were still pending for August and September as at 22 October. Fourthly, data were collected in aggregate format by age group, sex and clinical symptom and therefore no further analysis could be performed on specific age groups and clinical symptoms or sex ratio. Finally, to further assess the extent of the E30 upsurge in 2018 compared with earlier epidemics, longer data series would have been beneficial.

Specific prevention and control measures are not available for E30. Good hygiene practices such as frequent hand washing, avoidance of shared utensils, bottles or glasses and disinfection of contaminated surfaces (e.g. with diluted bleach solution) are recommended to prevent the spread of E30 from person to person. Further spread in the affected countries cannot be excluded and the non-affected countries or countries where enterovirus surveillance is lacking or not covering the whole population should remain vigilant for non-polio enterovirus outbreaks. It has been shown that considerable health resources can be saved by rapid detection of the virus [22], dissemination of information about the epidemics and a conservative approach to clinical management [26]. Differential diagnostics of viruses in meningitis cases may also prevent unnecessary use of antibiotics. As the epidemic is still ongoing in several countries, clinicians need to be made aware by national health authorities of this recent increase in E30 infections. Overall, EU/EEA countries have good coverage for detection of non-polio enteroviruses through their enterovirus surveillance systems [3]. For detection, technical recommendations are available from the European non-polio enterovirus network (ENPEN) [27]. It is also important for awareness raising that the countries share their data on enterovirus epidemics e.g. through the Epidemic Intelligence System (EPIS) and ENPEN to further understand the circulation and impact of non-polio enterovirus infections in Europe. As the E30 activity can last several years, it is relevant to consider preparedness for the 2019 season. 

This study has shown that the public health networks across EU/EEA can share their clinical and public health surveillance, and diagnostic laboratory data on emerging enterovirus infections ad hoc and in a timely fashion. Genetic analysis is ongoing and will shed light on whether the virus strain causing the increase in Europe is similar to previous years or novel. In addition, epidemiological investigations by the Member States will help understand the transmission patterns.

## Notes

**The EU/EAA Member State contributors:** Birgit Prochazka (National Polio Reference lab / AGES Vienna, Austria) Chloé Wyndham-Thomas (Epidemiology of infectious diseases, Epidemiology and public health, Sciensano, Brussels, Belgium) Marc Van Ranst (National Reference Centre for Enteroviruses including polioviruses et parechoviruses, Laboratory for Clinical and Epidemiological Virology, Rega Institute for Medical Research, KU Leuven, Leuven, Belgium) Irena Tabain (Virology Department Croatian Institute of Public Health, Croatia) Katerina Fabianova (National Institute of Public Health, Prague, Czech Republic) Sofie Midgley (Statens Serum Institut, Copenhagen, Denmark) Thea Kølsen Fischer (Statens Serum Institut, Copenhagen, Denmark; Nordsjaellands Hospital, Hilleroed, Denmark) Soile Blomqvist (National Institute for Health and Welfare, Helsinki, Finland) Sabine Diedrich (Robert Koch Institute, Berlin, Germany) Sindy Böttcher (Robert Koch Institute, Berlin, Germany) Kathrin Keeren (Robert Koch Institute, Berlin, Germany) Guðrún Erna Baldvinsdóttir (Virology, Landspitali University Hospital, Reykjavik, Iceland) Jeļena Storoženko (Riga East University Hospital, Latvian Centre of Infectious Diseases, National Microbiology Reference Laboratory, Latvia) Gatis Pakarna (Riga East University Hospital, Latvian Centre of Infectious Diseases, National Microbiology Reference Laboratory, Latvia) Natālija Zamjatina (Riga East University Hospital, Latvian Centre of Infectious Diseases, National Microbiology Reference Laboratory, Latvia) Tatjana Kolupajeva (Riga East University Hospital, Latvian Centre of Infectious Diseases, National Microbiology Reference Laboratory, Latvia) Kimberley Benschop (National Institute for Public Health and the Environment, the Netherlands; on behalf of VIRO-TypeNed) Natasa Berginc (National Laboratory of Health, Environment and Food - Laboratory for Public Health Virology, Slovenia) Susanne Dudman (Norwegian Institute of Public Health, Norway) Sanela Numanovic (Norwegian Institute of Public Health, Norway) Katherina Zakikhany (Public Health Agency of Sweden, Sweden) Elenor Hauzenberger (Public Health Agency of Sweden, Sweden) María Cabrerizo (Instituto de Salud Carlos III, Madrid, Spain) Richard Pebody (Public Health England, the United Kingdom) Jake Dunning (Public Health England, the United Kingdom) Alison Smith-Palmer (Health Protection Scotland, the United Kingdom) Catherine Moore (Public Health Wales Microbiology Cardiff, the United Kingdom) Laura Bubba (Public Health England, the United Kingdom; EUPHEM fellow, European Centre for Disease Prevention and Control) Heli Harvala (NHS Blood and Transplant, the United Kingdom)
